# A Simple Approach to Determine Single-Receiver Differential Code Bias Using Precise Point Positioning

**DOI:** 10.3390/s23198230

**Published:** 2023-10-03

**Authors:** Fenkai Zhang, Long Tang, Jiaxing Li, Xiangfeng Du

**Affiliations:** 1 School of Civil and Transportation Engineering, Guangdong University of Technology, Guangzhou 510006, China; 2112209048@mail2.gdut.edu.cn (F.Z.); 2112109050@mail2.gdut.edu.cn (J.L.); 2 School of Surveying and Remote Sensing Information Engineering, Guangdong Polytechnic of Industry and Commerce, Guangzhou 510550, China; dunac@163.com

**Keywords:** differential code biases, precise point positioning, carrier-to-code leveling, standard deviation

## Abstract

In this study, a precise single-receiver differential code bias (DCB) estimation method using the precise point positioning (PPP) model is presented. The first step is to extract the high-precision ionospheric observations, including DCB*s*, based on the PPP model. Then, the satellite DCB*s* are corrected using International GNSS Service (IGS) products. Lastly, the algorithm for the minimization of the standard deviation of vertical total electron content (VTECmstd) is employed to determine the value of receiver DCB. To check the method, GNSS data from more than 200 IGS stations around the globe on four days with various geomagnetic and solar activity circumstances are processed. The receiver DCB*s* are compared to those obtained using previous carried-to-code level (CCL) models. The experimental results show that, compared to the CCL model, the values of VTECmstd for most stations are significantly reduced, the mean number of stations with negative ionospheric measurements is reduced by 40% after correcting the receiver DCB*s*, and the mean error of estimated receiver DCB*s* is reduced by approximately 0.6 ns using the PPP model. These results suggest that this method can provide more high-precision receiver DCB estimation.

## 1. Introduction

Global navigation satellite system (GNSS) code observations are affected by hardware delays when they propagate in the internal channel of hardware, leading to the formation of differential code biases (DCB*s*) [1,2]. The magnitude of DCB*s* can be up to tens of nanoseconds, which can cause significant biases in ionospheric observations (namely total electron content (TEC)) and precise positioning. In order to obtain absolute and accurate TEC values and high-precision positions, both the satellite and receiver DCB*s* must be precisely calibrated [3,4]. Currently, the international GNSS service (IGS) and other ionosphere analysis centers can provide satellite DCB*s* products and part IGS receiver DCB*s* products [5]. For most GNSS stations, the receiver DCB*s* are still unknown.

Many methods were proposed to determine DCB*s*, which can be primarily divided into two categories. The first category employs mathematical models, such as spherical harmonic function [6], tomography [5], and generalized triangular series function [7], to express the ionospheric TEC and then estimate the ionospheric model parameters and DCB*s* simultaneously. The second category serves the TEC values in ionospheric observations as known values using external ionospheric TEC products, such as global ionospheric maps (GIM); then, the DCB*s* can be determined directly [8,9]. In addition, Ma and Maruyama (2003) presented a minimization of the standard deviation of the vertical total electron content (VTEC_mstd_) method to estimate the single-receiver DCB [10]. This method views the scattering of projected vertical TECs (VTECs) as the smallest when the receiver DCB is correctly calibrated. Among these DCB estimation methods, the ionospheric observations are commonly based on the carrier-to-code level (CCL) model, which is significantly subjected to leveling errors caused by the multipath effects and code noise [11].

Recently, the development of an uncombined precise point positioning (PPP) technique provides a way to extract high-precision ionospheric observations [12,13]. Compared to the CCL model, the ionospheric observations obtained using the PPP model are less affected by leveling errors [14]. Based on this, some researchers employed PPP ionospheric observations to estimate DCB*s*, and more accurate and reliable DCB estimations were obtained [15,16]. Furthermore, Wang et al. [17] indicate that using ionospheric observations based on a PPP fixed-ambiguity solution can further improve the DCB estimations compared to those of a PPP float-ambiguity solution. The involved estimation methods belong to the aforementioned first category, namely ionospheric modeling methods, which generally need complex models and extensive computations.

Here, we will employ the VTEC_mstd_ method to estimate the receiver DCB using ionospheric observations based on a PPP fixed-ambiguity solution. Compared to the methods of the first and second categories, the VTEC_mstd_ method is very simple and does not rely on external ionospheric TEC products. Due to the application of high-accuracy ionospheric observations, the results obtained via the estimated receiver DCB are expected be more accurate than the results derived using the CCL model. In the following sections, the methodologies for ionospheric observations extraction and the receiver DCB estimation are presented first. Then, the receiver DCB estimation results are evaluated. Finally, the conclusions are drawn.

## 2. Methods and Data

### 2.1. Methods

The basic code and phase observation with dual-frequency can be expressed as follows:

(1) $$\left\{\begin{array}{c} P_{i}=\rho +c\left(t_{r}-t^{S}\right)+\gamma_{i}I_{1}+T+d_{r,i}-d_{i}^{S}+e_{i}\;\;\;\;\;\;\;\;\;\;\;\;\;\;\;\;\;\;\\ L_{i}=\rho +c\left(t_{r}-t^{S}\right)-\gamma_{i}I_{1}+T+\lambda_{i}\left(N_{i}+b_{r,i}-b_{i}^{S}\right)+\varepsilon_{i} \end{array}\right.$$

where i (i = 1, 2) is the frequency number; $P_{i}$ and $L_{i}$ are the raw code and carrier phase measurements (m); ρ is the geometric range from receiver to satellite (m); c is the speed of light; $t_{r}$ and $t^{S}$ are the receiver satellite clock errors (s); $I_{1}$ is the ionospheric delay along the path of satellite and receiver at the first frequency (m); $\gamma_{i}$ = $f_{1}^{2}/f_{i}^{2}$ is a scaling factor related to frequency; T is troposphere delay (m); $d_{r,i}$ and $d_{i}^{S}$ are the receiver and satellite instrument biases of the code delays (m); $b_{r,i}$ and $b_{i}^{S}$ are the receiver and satellite instrument biases of the carrier phase delays (cycle); $\lambda_{i}$ is the phase wavelength (m); $N_{i}$ is the ambiguity of the carrier phase (cycle); and $e_{i}$ and $\varepsilon_{i}$ are the observational noise and the multipath effect for the code and phase observations.

Based on Equation (1), many geometry-related parameters can be eliminated by the geometry-free combination:

(2) $$\left\{\begin{array}{c} P_{I}=\left(\gamma_{2}-1\right)I_{1}+{DCB}^{s}-{DCB}_{r}+e_{I}\;\;\;\\ L_{I}=\left(\gamma_{2}-1\right)I_{1}+\theta +\varepsilon_{I}\;\;\;\;\;\;\;\;\;\;\;\;\;\;\;\;\;\;\;\;\;\;\;\;\;\;\;\;\;\;\; \end{array}\right.$$

where $P_{I}=P_{2}-P_{1}$ is the single difference between two code measurements; $\;L_{I}=L_{1}-L_{2}$ is the single difference between two phase measurements; ${DCB}^{s}=d_{1}^{s}-d_{2}^{s}$ and ${DCB}_{r}=d_{r,1}-d_{r,2}$ are the satellite and receiver DCB*s*; $\theta =\lambda_{1}\left(N_{1}+b_{r,1}-b_{1}^{S}\right)-\lambda_{2}\left(N_{2}+b_{r,2}-b_{2}^{S}\right)$;$\;and\;e_{I}$ and $\varepsilon_{I}$ are the differential observational noise and the multipath effect for the code and phase observations.

The CCL model first calculates the constant θ using the mean of $L_{I}-P_{I}$ in a continuous arc according to Equation (2). Then, the CCL ionospheric observation is obtained:

(3) $$\left\{\begin{array}{c} {TEC}_{CCL}=\frac{40.3}{f_{1}^{2}}I_{1,CCL}\;\;\;\;\;\;\;\;\;\;\;\;\;\;\;\;\;\;\;\;\;\;\;\;\;\;\;\;\;\;\;\;\;\;\;\;\;\;\;\;\;\;\;\;\;\;\;\;\;\;\;\;\;\;\;\;\;\;\;\;\;\;\;\;\;\;\;\;\;\;\;\;\;\;\;\;\;\;\;\;\;\;\;\;\;\;\;\;\;\;\;\;\;\;\;\\ I_{1,CCL}=\frac{1}{{(\gamma }_{2}-1)}\left(L_{I}-{\langle L_{I}-P_{I}\rangle }_{arc}\right)+\frac{1}{{(\gamma }_{2}-1)}({{DCB}_{r}-DCB}^{s}\;)+e_{lev} \end{array}\right.$$

where ${TEC}_{CCL}$ denotes the CCL ionospheric observations (TECU); $f_{1}$ is the first frequency; ${\langle L_{I}-P_{I}\rangle }_{arc}$ presents the mean of $L_{I}-P_{I}$ in a continuous arc; and $\;e_{lev}$ is the leveling error. In this work, we employed the averaging approach in the leveling technique [11].

Based on Equation (1), the form of the uncombined PPP model can be expressed as follows [13]:

(4) $$\left\{\begin{array}{c} P_{i}=\rho +c(\tilde{t_{r}}-\tilde{t^{S})}+T+\tilde{I_{1}}\;\;\;\;\;\;\;\;\;\;\;\;\;\;\;\;\;+e_{i}\\ L_{i}=\rho +c(\tilde{t_{r}}-\tilde{t^{S})}+T-\tilde{I_{1}}+\lambda_{i}\tilde{N_{i}}\;\;\;\;+\varepsilon_{i} \end{array}\right.$$

where

$$\left\{\begin{array}{c} \tilde{t_{r}}=c\cdot t_{r}+\frac{\gamma_{2}}{\gamma_{2}-1}d_{r,1}-\frac{1}{\gamma_{2}-1}d_{r,2}\\ \tilde{t^{S}}=c\cdot t^{S}+\frac{\gamma_{2}}{\gamma_{2}-1}d_{1}^{S}-\frac{1}{\gamma_{2}-1}d_{2}^{S}\\ \tilde{I_{i}}=\gamma_{i}\left(I_{1}+\frac{1}{1-\gamma_{2}}\left({DCB}_{r}-{DCB}^{s}\right)\right)\;\\ \tilde{N_{i}}=N_{i}+b_{r,i}-b_{i}^{S}-\frac{\gamma_{2}+1}{\gamma_{2}-1}(d_{r,1}-d_{1}^{S})+\frac{2}{\gamma_{2}-1}(d_{r,2}-d_{2}^{S}) \end{array}\right.$$

The parameter $\tilde{I_{1}}$ in Equation (4) can be estimated via the PPP ambiguity resolution [18]. Then, the ionospheric observations are obtained:

(5) $$\left\{\begin{array}{c} {TEC}_{ppp}=\frac{40.3}{f_{1}^{2}}I_{1,ppp}\;\;\;\;\;\;\;\;\;\;\;\;\;\;\;\;\;\;\;\;\;\;\;\;\;\;\;\;\;\;\;\;\;\;\;\;\;\;\;\;\;\;\;\;\;\;\\ I_{1,ppp}=\tilde{I_{1}}+\frac{1}{1-\gamma_{2}}\left({DCB}_{r}-{DCB}^{s}\right)+\varepsilon_{PPP} \end{array}\right.$$

where ${TEC}_{ppp}$ is the PPP ionospheric observation (TECU); and $\varepsilon_{PPP}$ is the estimation error, which is significantly smaller than the leveling error $e_{lev}$.

After extracting the ionospheric observation TEC in a slant direction, VTEC is obtained using a projected factor [6]. Here, both are calculated VTECs using the CCL model and PPP model. Then, the so-called VTEC_mstd_ approach is applied to estimate the corresponding receiver DCBs.

The VTEC_mstd_ algorithm is based on the viewpoint that the scattering of projected VTECs for all the satellite–receiver pairs observed in a single station is smallest when the related DCBs in ionospheric observations are correctly calibrated. All the satellite DCBs can be calibrated using IGS products. So, the receiver DCB is determined by searching a series of DCB candidates and finding the one that presents a minimum deviation of VTECs to their mean.

For a given DCB candidate, the standard deviation ($\sigma_{i}$) of VTECs to their mean is computed at epoch i,

(6) $$\sigma_{i}=\sqrt{\frac{1}{M_{i}}\cdot \sum_{m=1}^{M_{i}}{\left({VTEC}_{i}^{m}-\bar{{VTEC}_{i}}\right)}^{2}}$$

where $M_{i}$ is the number of ionospheric observations at epoch i. Then, the whole standard deviation during a day is obtained:

(7) $$\sigma_{all}=\sum_{i=1}^{N}\sigma_{i}$$

where N is the number of epochs during a day. The DCB candidate when $\sigma_{all}$ takes the minimum, namely VTEC_mstd_, is the final receiver DCB.

It should be noted that $\sigma_{all}$ is not directly computed using the VTEC values in a whole day due to the fact the VTEC varies with time. If so, the TEC variations will blend in $\sigma_{all}$, which may exert an adverse effect on DCB estimation.

### 2.2. Data

**Figure 1:**
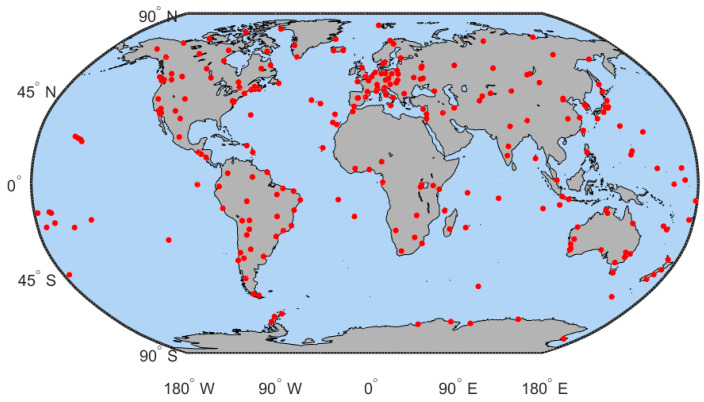
The distribution of used IGS stations.

Here, we only test the global positioning system (GPS) signals. The 30-s sampling GPS observation files from over 200 IGS stations around the globe (see Figure 1) were downloaded from the public website https://cddis.nasa.gov/archive/gnss/data/daily/ (accessed on 15 January 2023). Four days are selected on the basis of various geomagnetic and solar activity circumstances, namely DOY 19 and 50 from 2014 (high-solar activity year), and DOY 238 and 291 from 2018 (low-solar activity year), to assess the performance of the above-mentioned method (see Table 1). The F10.7 (solar radio flux, indication of solar activity), AE (auroral electrojet, indication of geomagnetic substorms), and Dst (indication of solar storms) were collected from the website https://omniweb.gsfc.nasa.gov/form/dx1.html (accessed on 15 January 2023). In the experiments, the candidate range for searching the correct receiver DCB is set to [–50,50] ns, and the searching step is set to 0.001 ns. In addition, the satellite elevation angle is set to 20 degrees to mitigate the impact of multipath effects on DCB estimation.

## 3. Results and Discussion

**Figure 2:**
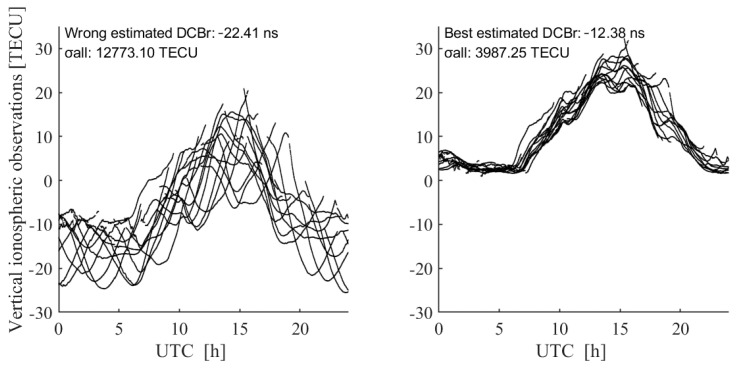
The vertical TEC corrected with incorrect receiver DCB value (**left**) and best receiver DCB estimation (**right**) at station ASCG on DOY 238, 2018.

Figure 2 presents the VTEC time series observed at the station ASCG on DOY 238, 2018. The satellite DCB*s* are identical and corrected using the IGS products for each panel. The receiver DCB is corrected with the value of −22.41 ns when $\sigma_{all}$ is 12773.10 TEC in the left panel, while the receiver DCB is corrected with the value of −12.38 ns when $\sigma_{all}$ is at its minimum (namely 3987.25 TECU) in the right panel. The figure distinctly illustrates that the correction with the erroneous DCB (−22.41 ns) amplifies the dispersion of the VTEC time series relative to the best DCB estimate (−12.38 ns) derived using the PPP approach. This result shows the validity of the VTEC_mstd_ method.

**Figure 3:**
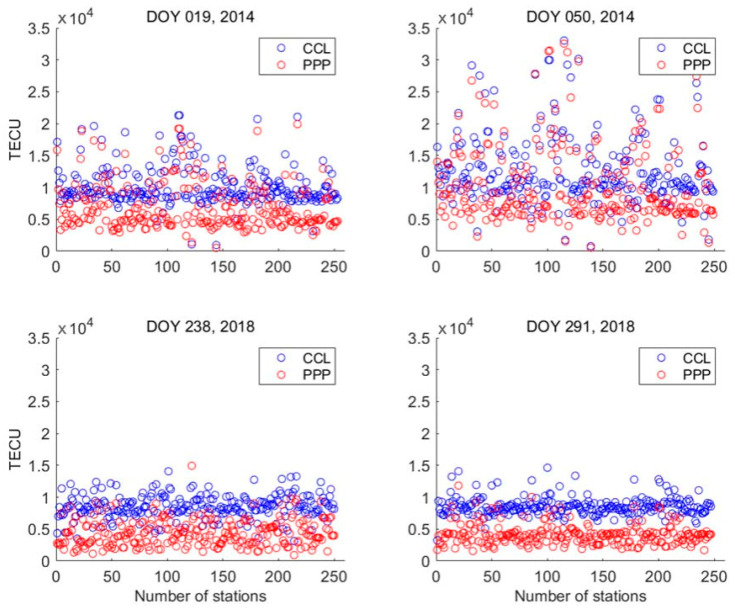
The values of VTEC_mstd_ (minimum $\sigma_{all}$) for all stations on different days using the CCL model (blue circles) and PPP model (red circles).

Figure 3 presents the values of VTEC_mstd_ for all stations on different days, using the CCL model (blue circles) and the PPP model (red circles). As shown in the figure, the magnitude of VTEC_mstd_ when using the PPP model is significantly smaller than that seen when using the CCL model for most stations, especially during the low-solar-activity year. The mean and its difference of VTEC_mstd_ on each day obtained using the PPP and CCL models are listed in Table 2. As shown in Table 2, the VTEC_mstd_ is large on the geomagnetic and solar activity days for both models. The large VTEC_mstd_ is reasonable because the ionospheric TEC and its gradient are large in the circumstances. Compared to the CCL model, the reduced mean VTEC_mstd_ exceeds 2500 TECU for each day using the PPP model. Obviously, the factor contributing to the reduction in VTEC_mstd_ is the high-precision PPP ionospheric observations, which are less affected by the leveling errors. This suggests that the estimated receiver DCB*s* using the PPP model are also less affected by the leveling errors.

**Figure 4:**
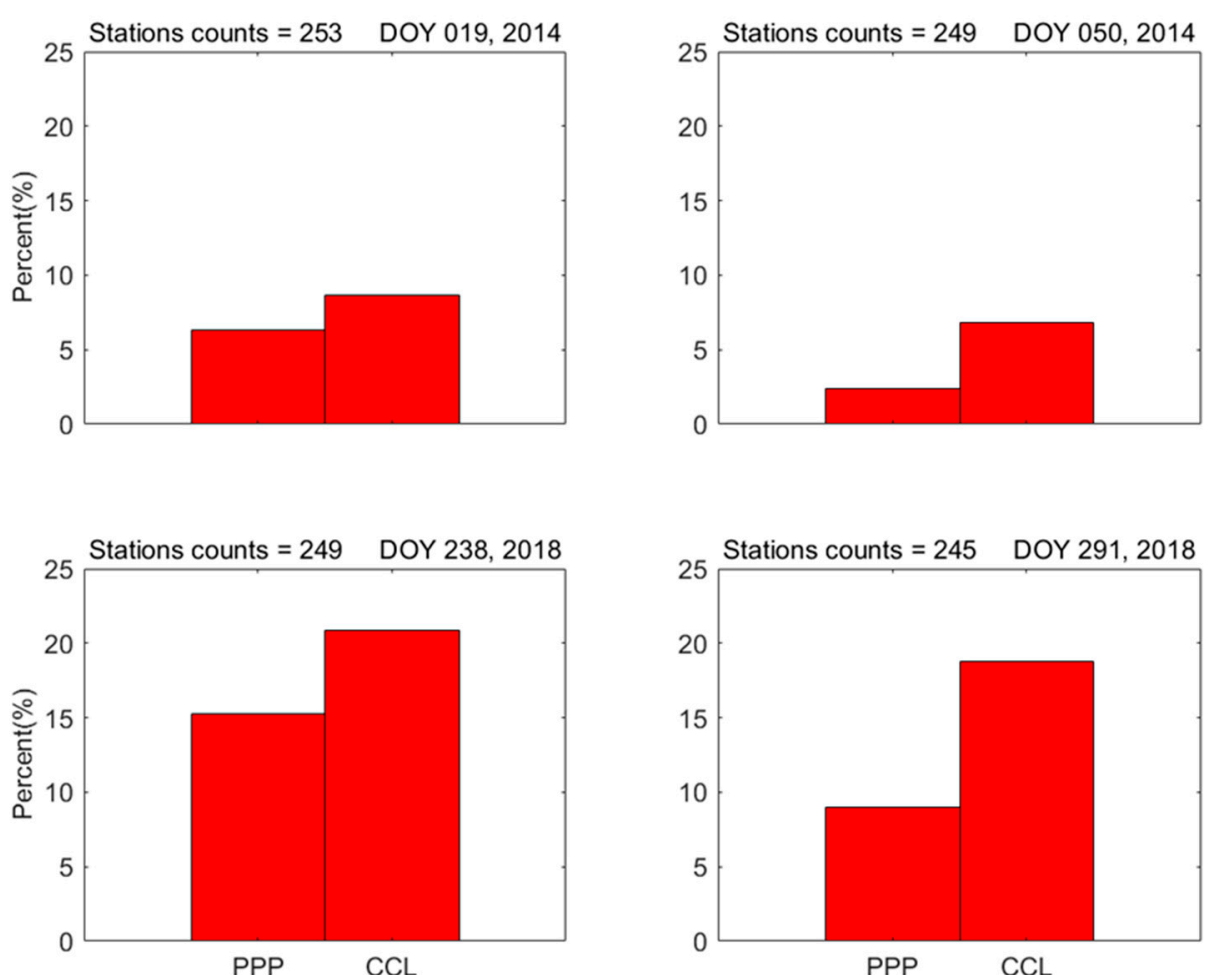
The percentages of stations including negative calibrated ionospheric observations using the CCL model and the PPP model.

Obviously, the time series of ionospheric observations would consist exclusively of positive values if the satellite and receiver DCB*s* were precisely calibrated. In situations where the CCL or PPP methods fail to accurately determine the receiver DCB values, negative TEC values may occur after correction with these estimates. Thus, this fact can serve as a metric with which to evaluate the precision of the estimated receiver DCB*s* [19,20]. Figure 4 presents the percentages of stations, including negatively calibrated ionospheric observations obtained using the CCL model and PPP model. As seen in Figure 4, the percentages obtained using the PPP model are significantly smaller than that derived using the CCL model on various days, especially on DOY 50, 2014, and DOY 291, 2018: the mean percentages for the four days are 8.24% and 13.80%, respectively. In other words, the mean number of stations with negative ionospheric measurements is reduced by 40% after correcting the receiver DCB*s*. This suggests that the receiver DCB*s* estimated using the PPP model are more precise. In addition, the percentages obtained during 2014 seem smaller than those from 2018. This is because the magnitude of ionospheric TEC is large during high solar-activity years, meaning that the negative ionospheric observation is not easily formed and vice versa.

**Figure 5:**
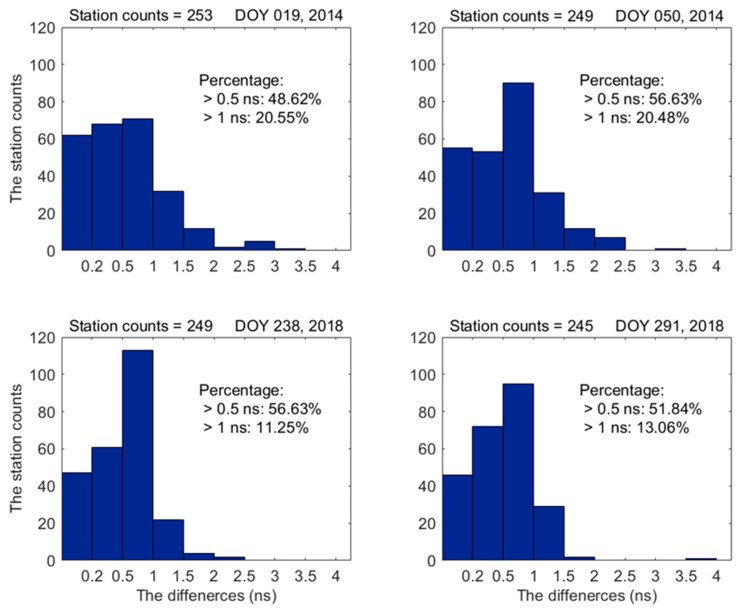
The distribution of difference values of estimated receiver DCB*s* between the PPP model and CCL model for all stations on different days.

The smaller values of VTECmstd and negative percentage suggest that the VTECmstd algorithm using the PPP model can enhance receiver DCB estimation accuracy. To assess the degree of improvement, we plot the distribution of difference values of estimated receiver DCB*s* between the PPP model and CCL model (|$∆{DCB}_{r}$|) for all stations on different days in Figure 5. In addition, the statistical results related to |$∆{DCB}_{r}$| are also listed in Table 3. As can be seen from Figure 5, the percentages of difference above 1 ns are 20.55%, 20.48%, 11.25%, and 13.06% on various days, respectively; as for |$∆{DCB}_{r}$| above 0.5 ns, the percentages are around 50%. As presented in Table 3, the means of difference on various days are approximately 0.6 ns. These results show that the receiver DCB estimation accuracy for many stations can be improved significantly.

**Figure 6:**
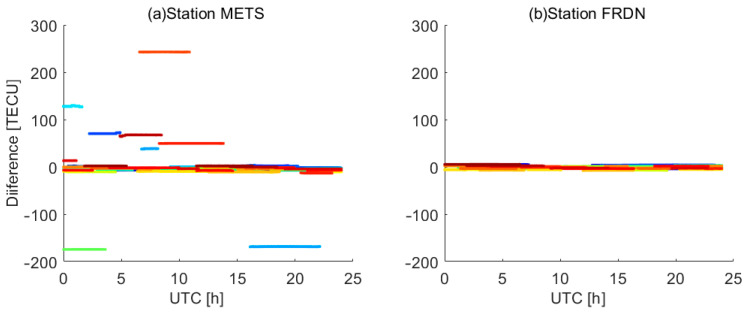
The differences of ionospheric observations between the PPP model and the CCL model at stations (**a**) METS and (**b**) FRDN. Each color corresponds to a satellite.

As shown in Table 3, the maximum values of |$∆{DCB}_{r}$| exceed 2 ns while the minimum values of |$∆{DCB}_{r}$| are near zero, suggesting that the differences vary from station to station. In order to elucidate the underlying factors, we conducted a detailed analysis of the stations METS and FRDN with the maximum (2.437 ns) and minimum (0.005 ns) differences on DOY 238, 2018, respectively. Figure 6 plots the differences in ionospheric observations between the PPP model and the CCL model at specific stations. As shown in the figure, the difference in ionospheric observations for the station METS ranges from −175 to 250 TECU, while the difference scope for station FRDN is within 10 TECU. That is to say, the ionospheric observations obtained using the CCL model at station METS (FRDN) have large (small) leveling errors. So, the value of |$∆{DCB}_{r}$| is related to the magnitude of leveling errors in ionospheric observations using the CCL model.

**Figure 7:**
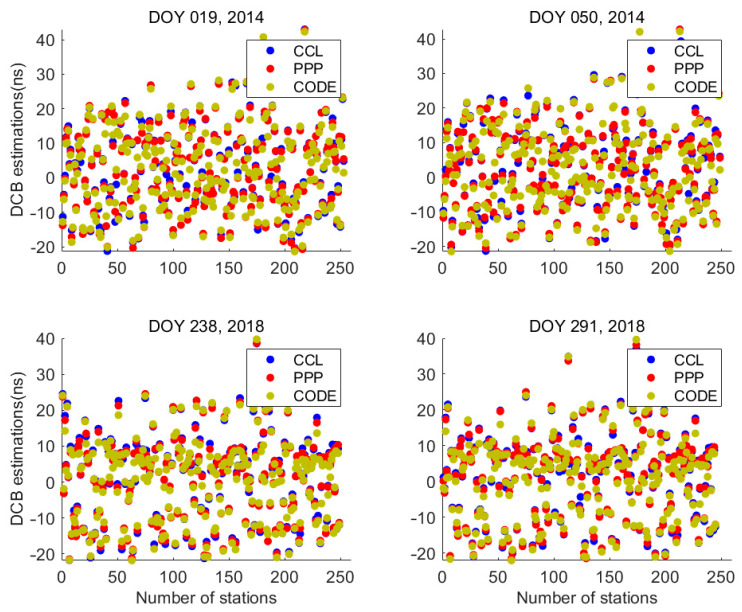
The values of receiver DCB estimations for all stations on different days about the CCL model (blue circles), PPP model (red circles) and CODE (yellow circles).

The receiver IGS DCB products are frequently used to assess the performance of DCB estimations [8,9]. Considering that the IGS products are also estimated using CCL measurements, we have not employed them as an absolute indicator that would demonstrate the superiority of the PPP method over the CCL. However, they can still serve as reference indicators to illustrate the consistency among estimation data and further affirm the effectiveness of the VTEC_mstd_ method. Figure 7 shows the receiver DCB*s* estimated via the PPP and CCL models and the DCB products provided by the Center for Orbit Determination in Europe (CODE, one of the IGS analysis centers). In addition, the statistical results of the differences between receiver DCB*s* estimated via the PPP and CCL models and the CODE receiver DCB*s* are also listed in Table 4. As the results show, the mean differences between the estimations from the two methods and the CODE DCB are approximately 0.5 ns. Furthermore, the receiver DCB estimations from the PPP model closely align with the CODE-DCBr values compared to those of the CCL model.

The above results show that the VTEC_mstd_ method using the PPP model can provide more high-precision receiver DCB estimation than using the CCL model on various geomagnetic and solar activity days. This can be attributed to the high-accuracy ionospheric observations obtained by the PPP model. However, as shown in Figure 4, the negative TEC values at some stations still exist after correcting receiver DCB using the PPP model, indicating the existence of estimation errors. The estimation errors are mainly caused by the algorithm of the VTEC_mstd_ method. To further improve the estimation accuracy of receiver DCB and eliminate the negative TEC values, multiple stations and a more complex algorithm are necessary. For example, Yasyukevich et al. [20] proposed the TuRBOTEC algorithm, employing bounded-variable least-squares fitting to ensure the non-negativity of TEC and effective DCB estimation.

Except for the VTEC_mstd_ method, some other single-receiver DCB estimation methods using external GIM products are presented in previous studies [8,9,21]. According to their results, the estimated receiver DCB*s* are consistent with the IGS products, which are similar to our counterparts. However, these algorithms may introduce errors from GIM products, particularly during ionospheric storm periods. Zhou et al. [16] employed the uncombined PPP model and the least-squares method to simultaneously estimate the vertical TEC and the satellite and receiver DCB*s*, eliminating the need for external products for estimation but requiring substantial computational effort.

## 4. Conclusions and Perspectives

In this study, we employ the VTEC_mstd_ method to estimate the receiver DCB using ionospheric observations based on a PPP fixed-ambiguity solution. To check the method, GPS data from more than 200 IGS stations around the globe on four days with various geomagnetic and solar activity circumstances are processed. The experimental results show that the method using the PPP model can provide DCB estimations of higher receiver precision than those obtained using the CCL model on various geomagnetic and solar activity days: the values of VTEC_mstd_ for most stations are significantly reduced, the mean number of stations with negative ionospheric measurements is reduced by 40% after correcting the receiver DCB*s*, and the mean error of estimated receiver DCB*s* is reduced by approximately 0.6 ns, which is roughly equivalent to 1.7 TECU. This is due to the fact that the ionospheric observations obtained using the PPP (CCL) model are less (more) affected by leveling errors. Here, only the VTEC_mstd_ method is investigated. In future studies, additional algorithms for estimating DCB using the ionospheric observations obtained via the PPP model should be explored to further assess their performance.

## Figures and Tables

**Table 1:** The solar F10.7, AE, and Dst index (daily averaged value) for the selected days.

Year	DOY	F10.7	AE	Dst Index (nT)
2014	019	123.4	26	8
2014	050	154.2	443	−66
2018	238	72.6	692	−104
2018	291	69.0	20	−2

**Table 2:** The mean and its difference of VTEC_mstd_ (minimum $\sigma_{all}$) on each day using the PPP model and CCL model.

Year	DOY	PPP (TECU)	CCL (TECU)	PPP–CCL (TECU)
2014	019	6956.09	10,199.93	−3243.84
2014	050	10,193.44	12,752.69	−2559.24
2018	238	4382.26	8829.41	−4447.16
2018	291	4136.07	8700.66	−4564.58

**Table 3:** The statistical results related to the difference values of estimated receiver DCB*s* between the PPP model and CCL model.

Year	DOY	Mean (ns)	Minimum (ns)	Maximum (ns)
2014	019	0.640	0.002	3.263
2014	050	0.657	0.004	3.174
2018	238	0.584	0.005	2.437
2018	291	0.562	0.003	3.753

**Table 4:** The statistical results of the differences between the receiver DCB*s* estimated via the PPP and CCL models and the corresponding CODE products.

Year	DOY	Mean (ns)		Minimum (ns)		Maximum (ns)	
		CCL	PPP	CCL	PPP	CCL	PPP
2014	019	−0.686	−0.491	−5.349	−5.034	6.308	4.252
2014	050	−0.348	−0.347	−5.357	−5.697	7.640	7.019
2018	238	−0.596	−0.502	−5.074	−4.862	3.041	2.954
2018	291	−0.617	−0.587	−4.992	−4.660	3.693	2.117

## Data Availability

Not applicable.

## References

[1] Sardon E., Rius A., Zarraoa N. (1994). Estimation of the transmitter and receiver differential biases and the ionospheric total electron content from Global Positioning System observations. Radio Sci..

[2] Håkansson M., Jensen A.B., Horemuz M., Hedling G. (2017). Review of code and phase biases in multi-GNSS positioning. GPS Solut..

[3] Jensen A.B.O., Ovstedal O., Grinde G. Development of a regional ionosphere model for Norway. Proceedings of the International Technical Meeting of the Satellite Division of the Institute of Navigation.

[4] Montenbruck O., Hauschild A. Code biases in multi-GNSS point positioning. Proceedings of the ION-ITM-2013.

[5] Hernández-Pajares M., Juan J.M., Sanz J., Orus R., Garcia-Rigo A., Feltens J., Komjathy A., Schaer S.C., Krankowski A. (2009). The IGS VTEC maps: A reliable source of ionospheric information since 1998. J. Geod..

[6] Schaer S. (1999). Mapping and Predicting the Earth’s Ionosphere Using the Global Positioning System.

[7] Li Z., Yuan Y., Li H., Ou J., Huo X. (2012). Two-step method for the determination of the differential code biases of COMPASS satellites. J. Geod..

[8] Arikan F., Nayir H., Sezen U., Arikan O. (2008). Estimation of single station interfrequency receiver bias using GPS-TEC. Radio Sci..

[9] Keshin M. (2012). A new algorithm for single receiver DCB estimation using IGS TEC maps. GPS Solut..

[10] Ma G., Maruyama T. (2003). Derivation of TEC and estimation of instrumental biases from GEONET in Japan. Ann. Geophys..

[11] Ciraolo L., Azpilicueta F., Brunini C., Meza A., Radicella S.M. (2007). Calibration errors on experimental slant total electron content (TEC) determined with GPS. J. Geod..

[12] Zhang B., Ou J., Yuan Y., Li Z. (2012). Extraction of line-of-sight ionospheric observables from GPS data using precise point positioning. Sci. China Earth Sci..

[13] Xiang Y., Gao Y., Shi J., Xu C. (2019). Consistency and analysis of ionospheric observables obtained from three precise point positioning models. J. Geod..

[14] Zhang B. (2016). Three methods to retrieve slant total electron content measurements from ground-based GPS receivers and performance assessment. Radio Sci..

[15] Liu T., Zhang B., Yuan Y., Li Z., Wang N. (2019). Multi-GNSS triple-frequency differential code bias (DCB) determination with precise point positioning (PPP). J. Geod..

[16] Zhou P., Nie Z., Xiang Y., Wang J., Du L., Gao Y. (2020). Differential code bias estimation based on uncombined PPP with LEO onboard GPS observations. Adv. Space Res..

[17] Wang J., Huang G., Zhou P., Yang Y., Zhang Q., Gao Y. (2020). Advantages of Uncombined Precise Point Positioning with Fixed Ambiguity Resolution for Slant Total Electron Content (STEC) and Differential Code Bias (DCB) Estimation. Remote Sens..

[18] Li P., Cui B., Hu J., Liu X., Zhang X., Ge M., Schuh H. (2022). PPP-RTK considering the ionosphere uncertainty with cross-validation. Satell. Navig..

[19] Yasyukevich Y.V., Mylnikova A.A., Polyakova A.S. (2015). Estimating the total electron content absolute value from the GPS/GLONASS data. Results Phys..

[20] Yasyukevich Y.V., Mylnikova A., Vesnin A. (2020). GNSS-based non-negative absolute ionosphere total electron content, its spatial gradients, time derivatives and differential code biases: Bounded-variable least-squares and taylor series. Sensors.

[21] Li M., Yuan Y., Wang N., Liu T., Chen Y. (2018). Estimation and analysis of the short-term variations of multi-GNSS receiver differential code biases using global ionosphere maps. J. Geod..

